# Dental Age Estimation from Panoramic Radiographs: A Comparison of Orthodontist and ChatGPT-4 Evaluations Using the London Atlas, Nolla, and Haavikko Methods

**DOI:** 10.3390/diagnostics15182389

**Published:** 2025-09-19

**Authors:** Derya Dursun, Rumeysa Bilici Geçer

**Affiliations:** Department of Orthodontics, Hamidiye Faculty of Dental Medicine, University of Health Sciences, Istanbul 34668, Turkey; derya.dursun@sbu.edu.tr

**Keywords:** artificial intelligence, ChatGPT, Haavikko, large language models, London Atlas, Nolla, panoramic radiographs

## Abstract

**Background:** Dental age (DA) estimation, which is widely used in orthodontics, pediatric dentistry, and forensic dentistry, predicts chronological age (CA) by assessing tooth development and maturation. Most methods rely on radiographic evaluation of tooth mineralization and eruption stages to assess DA. With the increasing adoption of large language models (LLMs) in medical sciences, use of ChatGPT has extended to processing visual data. The aim of this study, therefore, was to evaluate the performance of ChatGPT-4 in estimating DA from panoramic radiographs using three conventional methods (Nolla, Haavikko, and London Atlas) and to compare its accuracy against both orthodontist assessments and CA. **Methods:** In this retrospective study, panoramic radiographs of 511 Turkish children aged 6–17 years were assessed. DA was estimated using the Nolla, Haavikko, and London Atlas methods by both orthodontists and ChatGPT-4. The DA–CA difference and mean absolute error (MAE) were calculated, and statistical comparisons were performed to assess accuracy and sex differences and reach an agreement between the evaluators, with significance set at *p* < 0.05. **Results:** The mean CA of the study population was 12.37 ± 2.95 years (boys: 12.39 ± 2.94; girls: 12.35 ± 2.96). Using the London Atlas method, the orthodontists overestimated CA with a DA–CA difference of 0.78 ± 1.26 years (*p* < 0.001), whereas ChatGPT-4 showed no significant DA–CA difference (0.03 ± 0.93; *p* = 0.399). Using the Nolla method, the orthodontist showed no significant DA–CA difference (0.03 ± 1.14; *p* = 0.606), but ChatGPT-4 underestimated CA with a DA–CA difference of −0.40 ± 1.96 years (*p* < 0.001). Using the Haavikko method, the evaluators underestimated CA (orthodontist: −0.88; ChatGPT-4: −1.18; *p* < 0.001). The lowest MAE for ChatGPT-4 was obtained when using the London Atlas method (0.59 ± 0.72), followed by Nolla (1.33 ± 1.28) and Haavikko (1.51 ± 1.41). For the orthodontists, the lowest MAE was achieved when using the Nolla method (0.86 ± 0.75). Agreement between the orthodontists and ChatGPT-4 was highest when using the London Atlas method (ICC = 0.944, r = 0.905). **Conclusions:** ChatGPT-4 showed the highest accuracy with the London Atlas method, with no significant difference from CA for either sex or the lowest prediction error. When using the Nolla and Haavikko methods, both ChatGPT-4 and the orthodontist tended to underestimate age, with higher errors. Overall, ChatGPT-4 performed best when using visually guided methods and was less accurate when using multi-stage scoring methods.

## 1. Introduction

Chronological age (CA) refers to the time elapsed since an individual’s birth and serves as a fundamental parameter in legal, educational, and clinical decision-making processes [1,2]. However, in cases where official birth records are missing, or their reliability is questionable—such as among refugee children, undocumented individuals, or forensic cases—alternative methods for age estimation become necessary [3,4]. In such instances, dental age (DA) estimation emerges as a practical and biologically grounded approach. DA estimation involves evaluating the development and maturation of dentition to predict an individual’s chronological age [5]. It is commonly applied in orthodontics for determining the optimal timing for treatment, in pediatric dentistry for growth monitoring, and in forensic dentistry and anthropology for identification and age assessment [3,4]. Several methods have been proposed to estimate DA, with these being primarily based on the degree of mineralization and eruption stages of teeth visible in radiographs [4]. It is widely accepted that calcification-based assessments offer greater reliability than eruption-based evaluations when estimating age [6]. Compared to skeletal or sexual maturity indicators, tooth calcification is considered to be less affected by environmental factors such as nutrition, metabolism, and genetic variation, making it a preferred method in age estimation practices [4,5]. Radiographic age estimation techniques are generally categorized into three main approaches: atlas-based comparisons, stage-based scoring systems, and formulaic methods derived from direct measurements [2]. The Nolla method [7], the Haavikko method, and the London Atlas method [8] are among the most frequently used radiographic techniques for assessing calcification stages.

The Nolla method, developed in 1960, is used to evaluate the calcification stage of permanent teeth based on a 10-stage scale [7]. Each tooth is given a numerical value corresponding to its developmental stage, and the total score is compared with standardized reference tables to estimate the individual’s dental age [1,4].

The Haavikko method, originally formulated through an analysis of panoramic radiographs from a cohort of Finnish children aged 2 to 13 years, incorporates both the calcification and eruption stages of selected permanent teeth [9]. As a scoring-based approach, it is considered more detailed than purely eruption-focused systems [2,9,10].

In contrast, the London Atlas method provides an evidence-based, graphical reference that combines both tooth development and alveolar eruption stages. Introduced by AlQahtani et al. [8] in 2014 it consists of 31 chronological age categories, spanning from 28 weeks in utero to 23 years of age. Unlike many other systems, the London Atlas method does not differentiate between sexes and is widely accessible in multiple languages through an online platform [8]. Although radiographic DA estimation methods have been reliably used for decades, they are not without their limitations. Many traditional techniques are time-consuming and require labor-intensive, manual assessment, which introduces variability across examiners. They also involve subjective interpretation, as different evaluators may assign different developmental stages to the same radiograph. Furthermore, their accuracy can be compromised by population-specific differences—such as ethnicity, environmental factors, and dietary habits—leading to inconsistent performance across diverse cohorts [11,12].

As artificial intelligence (AI) technologies continue to evolve, their applications in healthcare—particularly in the fields of medicine and dentistry—have expanded rapidly. One prominent example is the Chat Generative Pretrained Transformer (ChatGPT), a large language model developed by OpenAI, which has been widely utilized in various clinical and educational contexts since its public release [13]. In the medical and dental domains, ChatGPT has been effectively employed in a range of text-based tasks, including educating patients, summarizing the medical literature, simplifying explanations of diagnoses and treatment options, generating academic content, and simulating patient–provider interactions [14]. Collectively, these applications suggest that ChatGPT has the potential to support clinicians in communication-related tasks and enhance patient engagement, although further improvements are still necessary. While the image processing capabilities of ChatGPT were introduced with the launch of version 4.0 in late 2023 and remain in the early stages of development, the use of ChatGPT in image-based diagnostic tasks—such as radiographic interpretation—is relatively new and has only been explored in a limited number of studies [15]. Although the model now includes image input functionality, it has not yet been broadly validated for radiographic evaluation or diagnostic accuracy in clinical settings. One of the few studies addressing this topic evaluated the diagnostic performance of ChatGPT-4 in determining the labiolingual position of maxillary impacted canines and detecting root resorption in adjacent incisors using panoramic radiographs [15]. The retrospective analysis found that ChatGPT-4 exhibited low diagnostic accuracy in these tasks, with a reported accuracy of 37.1% for canine localization and 46.0% for resorption detection, indicating its unsuitability for clinical use [15]. In another study, Silva et al. [16] investigated ChatGPT’s diagnostic performance in detecting dental lesions using panoramic radiographs. They concluded that its accuracy was insufficient for clinical application, particularly for tasks requiring high diagnostic sensitivity.

Given these findings, investigating the potential of general-purpose multimodal AI models such as ChatGPT for image-based diagnostic tasks—particularly for estimating DA from panoramic radiographs—represents a novel direction in the literature. Accordingly, the aim of this study is to evaluate the performance of ChatGPT-4 in DA estimation using three established methods (Nolla, Haavikko, and the London Atlas) and to compare its evaluations with those of orthodontists and the CAs of the individuals assessed. In this way, this study offers a direct comparative analysis of AI and clinician performance, encompassing both visual assessment and stage-scoring, system-based methods. The null hypothesis states that there is no statistically significant difference among the estimations of ChatGPT-4 and orthodontists and the CA.

## 2. Materials and Methods

### 2.1. Sampling Method

This retrospective cross-sectional study included panoramic radiographs from 511 patients aged between 6 and 17 years, who presented to the Department of Orthodontics at the University of Health Sciences between January 2024 and July 2025. All radiographs were obtained using a digital panoramic imaging device (PCH-2500, Vatech, Hwaseong, Gyeonggi-do, Republic of Korea) under standardized conditions. The study protocol was reviewed and approved by the Hamidiye Non-Interventional Clinical Research Ethics Committee of the University of Health Sciences (approval Number: 2025/12/40, 29 May 2025). A priori sample size estimation was performed based on the approach of Gelbrich et al. [17]. A power analysis indicated that a minimum of 389 individuals would be required to achieve 90% power for detecting a clinically relevant bias of ±0.25 years (SD = 1.5 years), corresponding to an effect size of d = 0.17, at a significance level of α = 0.05. As this study included 511 participants, the sample size was considered sufficient. No data were missing or excluded from the analysis.

### 2.2. Selection Criteria

Only patients with clearly documented birth dates and dates of panoramic radiograph acquisition were included to enable accurate calculation of CA. Panoramic radiographs with inadequate image quality, motion artifacts, a visible history of surgical procedures involving the jaw, or clear developmental dental anomalies were excluded. Further exclusion criteria included the presence of any syndrome, systemic disease affecting dental development, previous orthodontic or endodontic treatment, and radiographic evidence of jaw pathology (such as cysts or tumors) or congenital tooth agenesis, except for the third molars. All radiographs were obtained using a digital panoramic radiography system under standard clinical imaging protocols.

### 2.3. Chronological and Dental Age Estimation

**Figure 1:**
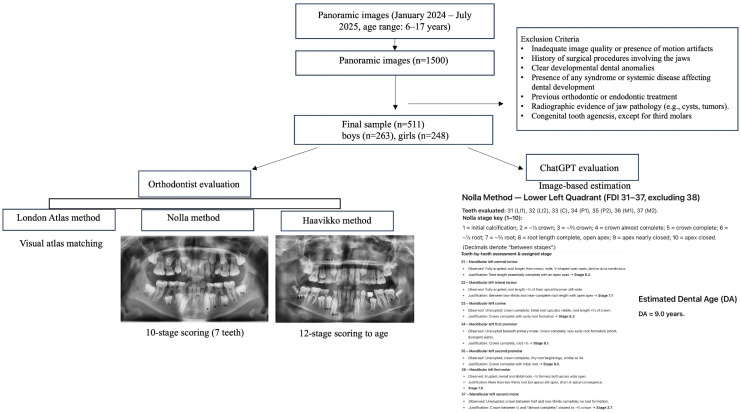
Flowchart illustrating the workflow for dental age estimation from panoramic radiographs.

The patients’ CA was determined using the decimal system by applying the following equation in Microsoft Excel 2013 (Microsoft, Redmond, WA, USA): [(the date when the panoramic radiograph was obtained) − (the documented birth date)]/365.25 [2]. Figure 1 shows the workflow for dental age estimation using the London Atlas, Nolla, and Haavikko methods.

The orthodontist assessments were conducted by two specialists with 14 and 7 years of clinical experience, respectively. Before beginning the main study, 50 randomly selected panoramic radiographs were evaluated by each examiner to calibrate both the methods and the examiners. To prevent discrepancies and achieve a single final decision, the orthodontists reviewed and discussed the evaluation methods and criteria reported in the literature in advance, reaching a consensus on their application. Each orthodontist then independently evaluated the panoramic radiographs, recording their results in separate Word documents. Inter-rater agreement for the final numerical dental age estimates was quantified using the intraclass correlation coefficient (ICC), which exceeded 0.90 and indicated excellent reliability.

In the Nolla method, the crown and root development of seven permanent teeth in the lower left quadrant (excluding third molars) are categorized into ten developmental stages. Each tooth is assigned a score from 1 to 10 based on its calcification status. Each tooth is assigned a numerical value reflecting its calcification progress, and if a tooth falls between two stages, intermediate values (e.g., 0.2, 0.5, 0.7) can be applied to capture this variance. The scores for all teeth are summed—often calculated separately and then compared to standardized reference tables, which are adjusted for sex and the presence of third molars, to estimate the individual’s dental age (DA) [1,4,7].

In the Haavikko method, DA estimation was performed based on Haavikko’s radiographic maturation criteria [10]. Each tooth’s development stage was then matched with age values according to Haavikko’s sex-specific reference tables. In individuals with a CA up to 10 years, teeth 47, 46, (16), 44, and 41 were evaluated, whereas in those older than 10 years, teeth 47, 44, 13, and 43 were assessed [2]. The individual age values derived from all eligible teeth were averaged to determine the final DA. Teeth with completed root formation—indicated by closed apices—were excluded from the calculation to maintain accuracy in reflecting active developmental stages.

In the London Atlas method, dental development and eruption stages are visually assessed using a color-coded diagram interface provided in a software tool (https://www.qmul.ac.uk/dentistry/atlas/software-app/, accessed on 1 April 2025). Users can select gender options (mixed, male, or female) and then determine the developmental stage of each tooth by comparing them to standard illustrations derived from the Moorrees classification method [18]. All selected parameters contribute to estimating the overall DA [8].

The same panoramic radiographs evaluated by the orthodontists were also assessed using the multimodal version of ChatGPT-4 (https://chat.openai.com) through the paid subscription service (ChatGPT Plus), which allows for image-based input analysis. Each radiograph was submitted independently without providing any demographic data or chronological age information.

Three separate prompts were developed for each of the DA estimation methods—Nolla, Haavikko, and the London Atlas. These prompts were designed and refined using Iterative Prompt Calibration (IPC) to optimize the model’s comprehension and output quality [15]. To further enhance accuracy and transparency in decision-making, the Chain of Thought (CoT) prompting technique was applied, encouraging the model to reason step-by-step before arriving at its final DA estimation [15]. The prompt development stages for each method are presented in Table 1.

All evaluations were performed on the same day by a single researcher using a consistent hardware setup. To eliminate session memory effects, a new ChatGPT-4 session was initiated for each evaluation. All evaluation results were systematically documented and stored in Microsoft Excel (Microsoft Corporation, Redmond, WA, USA) for subsequent statistical analyses.

### 2.4. Statistical Analysis

The DA–CA difference was calculated by subtracting the CA from the DA. A positive value indicated age overestimation, while a negative value indicated underestimation. The mean and standard deviation (SD) of DA, the DA–CA difference, and the mean absolute error (MAE) were calculated for each evaluator (orthodontist and ChatGPT-4) across the three methods (Nolla, Haavikko, and London Atlas). The MAE, accepted as the mean prediction error, was calculated to determine the estimation accuracy independently of DA–CA difference [1]. Normality assumptions were verified using appropriate tests, and since the data met these assumptions, parametric analyses were employed. A paired samples *t*-test was then used to compare the DA and CA within each evaluator group in order to determine whether the estimations significantly differed from the CA. An independent samples *t*-test was performed to compare DA–CA differences between males and females. The intra-class correlation coefficient (ICC) and Spearman’s correlation coefficients were computed to assess the agreement and association between the CA and DA, with ICC values below 0.4 considered poor, between 0.4 and 0.75 considered fair to good, and above 0.75 considered excellent [19]. Finally, a paired samples *t*-test was employed to compare the DA–CA differences between the orthodontists and ChatGPT-4 for each method. A *p*-value of less than 0.05 was considered statistically significant. All data were analyzed using IBM SPSS Statistics version 25 (IBM Corp., Armonk, NY, USA).

## 3. Results

The overall mean CA of the study population was 12.37 ± 2.95 years, with the mean CA being 12.39 ± 2.94 years for boys and 12.35 ± 2.96 years for girls.

As presented in Table 2, the differences between the mean DA and CA varied across the three estimation methods and evaluators. For the London Atlas method, the orthodontists significantly overestimated age (0.78 ± 1.26 years, *p* < 0.001), whereas ChatGPT-4’s estimations showed no significant difference from CA (0.03 ± 0.93 years, *p* = 0.399). In the Nolla method, the orthodontist estimates did not differ from CA (0.03 ± 1.14 years, *p* = 0.606), while ChatGPT-4 significantly underestimated them by 0.40 ± 1.96 years (*p* < 0.001). In the Haavikko method, both evaluators underestimated CA, with the orthodontists underestimating it by 0.88 ± 1.49 years and ChatGPT-4 by 1.18 ± 1.70 years (both *p* < 0.001).

**Figure 2:**
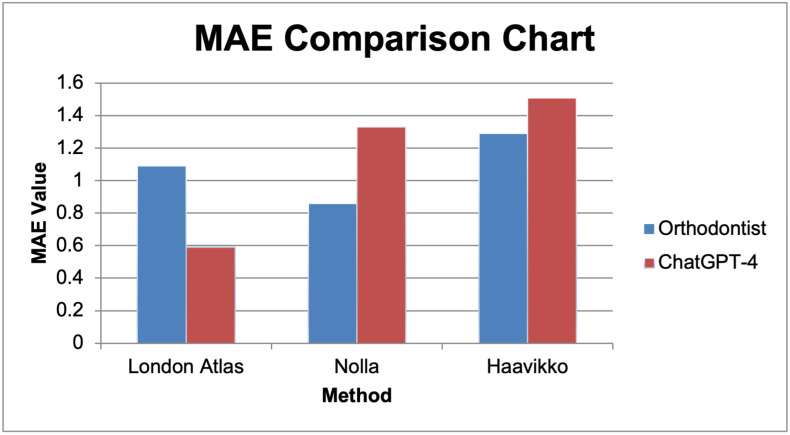
Mean absolute error (years) across methods for orthodontist vs. ChatGPT-4.

When the MAE values were compared, ChatGPT-4 produced the lowest error rates using the London Atlas method (0.59 ± 0.72 years), which indicates a higher level of accuracy for this approach. In the Nolla method, orthodontists had lower MAE (0.86 ± 0.75 years) than ChatGPT-4 (1.33 ± 1.28 years). In the Haavikko method, both evaluators showed higher MAE than in the other methods, with ChatGPT-4 slightly exceeding the orthodontists (Figure 2).

As shown in Table 3, the differences between the mean DA and CA varied by sex, estimation method, and evaluator. In boys, the results from the London Atlas method revealed significant overestimation by the orthodontists (0.80 ± 1.49 years; *p* < 0.001), while ChatGPT-4 showed no significant difference (0.02 ± 0.96 years; *p* = 0.749). With the Nolla method, orthodontist estimates did not differ significantly from CA (−0.01 ± 1.20 years; *p* = 0.897), whereas ChatGPT-4 significantly underestimated CA (−0.34 ± 1.89 years; *p* = 0.003). In the Haavikko method, both evaluators significantly underestimated CA (*p* < 0.001).

When MAE values were compared, ChatGPT-4 achieved the lowest MAE using the London Atlas method for both boys (0.59 ± 0.76 years) and girls (0.60 ± 0.68 years). For the Nolla and Haavikko methods, the orthodontists generally achieved lower MAEs than ChatGPT-4, except when using the Haavikko method for boys, where the difference was minimal.

Table 4 shows the differences between the DA and CA estimates provided by the orthodontists and ChatGPT-4 for each method. In the London Atlas method, orthodontist estimates were significantly higher than those of ChatGPT-4 (mean difference 0.75 ± 1.39 years, *p* < 0.001). A similar pattern was observed in the Nolla method, where orthodontist estimates exceeded ChatGPT-4’s by 0.42 ± 2.17 years (*p* < 0.001) and, in the Haavikko method, with a difference of 0.31 ± 1.24 years (*p* < 0.001). Thus, across all three methods, the orthodontists tended to produce higher DA estimates, with the greatest discrepancy occurring when using the Nolla method. However, the mean discrepancies remained small (0.31–0.75 years), although the standard deviations (1.24–2.17 years) indicate that individual-level differences may occasionally approach 2 years.

Table 5 presents the agreement between the DA estimates and CA for each method, as assessed by the orthodontists and ChatGPT-4. For the London Atlas method, agreement between the orthodontists and ChatGPT-4 was high; in the Nolla method, agreement remained good but comparatively lower; and for the Haavikko method, agreement was high as well. Thus, all three methods demonstrated excellent agreement (ICC > 0.75) according to the literature [19], although the Nolla method showed relatively lower rates of consistency than the London Atlas and Haavikko methods.

## 4. Discussion

Recent studies have evaluated ChatGPT and other large language models (LLMs) in dentistry [20,21], mostly focusing on text-based applications such as patient information and education [13,22,23,24]. However, their ability to perform complex tasks like visual interpretation remains limited [16]. The image input capability of ChatGPT-4, introduced in September 2023, enables radiographic interpretation, but its accuracy and reliability remain unvalidated. DA estimation is crucial in pediatric dentistry and orthodontics. Radiographic methods are preferred for their simplicity and reliability, but multi-stage scoring can be time-consuming [2,25,26]. Deep learning approaches may improve efficiency and objectivity in forensic and clinical contexts [27].

Previous studies have shown inconsistent results regarding the accuracy of the Nolla method in Turkish populations. Nur et al. [28] and Miloglu et al. [29] reported an underestimation tendency, particularly in girls, whereas Altunsoy et al. [30] found no significant differences in adolescents aged 12–16.9 years. By contrast, Koc et al. [1] reported mean differences of −0.41 ± 1.018 years for boys and −0.57 ± 1.027 years for girls, with no statistically significant difference between CA and DA, and concluded that the Nolla method could be reliably applied to Eastern Turkish children. In the present study, orthodontist assessment using the Nolla method revealed no significant differences between CA and DA in either sex (−0.01 ± 1.20 years in boys; 0.06 ± 1.08 years in girls), leading to the conclusion that the method is applicable for Eastern Turkish children. These variations across studies may reflect regional heterogeneity within Turkey, as environmental and climatic factors can influence dental development. Regarding the Haavikko method, earlier studies consistently demonstrated underestimation in Turkish children, with mean differences ranging between −0.30 and −0.70 years [2,25]. In the present study, comparable results were observed, with orthodontist estimations yielding −0.59 ± 1.38 years for boys and −1.19 ± 1.54 years for girls—both representing significant underestimations compared with chronological age. In the orthodontist assessment using the London Atlas method, Sezer et al. [2] reported no statistically significant differences between CA and DA in Turkish boys (0.03 ± 0.54 years), finding a small but statistically significant overestimation in girls instead (0.15 ± 0.60 years). In the present study, the orthodontists’ assessments produced larger mean differences—0.80 ± 1.49 years in boys and 0.79 ± 1.06 years in girls—both of which were statistically significant. By contrast, ChatGPT-4 yielded estimates much closer to the CA in both sexes (0.02 ± 0.96 years for boys and 0.05 ± 0.91 years for girls), with no statistically significant differences observed, indicating superior agreement with the true chronological ages compared to orthodontist assessments. Additionally, the London Atlas method in the present study yielded DA estimates by ChatGPT-4 that were very close to the CA for both boys and girls, with no statistically significant differences observed. By contrast, both the Nolla and Haavikko methods demonstrated a consistent underestimation tendency across sexes.

Yavuz et al. [26] employed convolutional neural networks (CNNs) for DA estimation, developing specifically trained systems. Such models, when trained on large datasets, can achieve high accuracy in visual recognition and classification tasks. In our study, however, we utilized ChatGPT-4, a language model with no prior training for dental age estimation. The model’s evaluations were guided solely by our instructions and the application of the CoT prompting technique, which encourages step-by-step reasoning. This approach may explain ChatGPT-4’s superior performance in the visually based London Atlas method, which relies on direct comparison with reference images. In this method, the model produced DA estimates closely aligned with CA, showing no statistically significant difference. Conversely, in methods based on numerical scoring of mineralization stages, such as the Nolla and Haavikko techniques, ChatGPT-4’s performance was lower.

Prompt optimization is critical for improving LLM performance in domain-specific tasks, with techniques such as CoT and IPC shown to enhance reasoning accuracy and consistency [31,32]. Salmanpour et al. [15] applied these methods in radiographic tasks, and in the present study, IPC was used for reproducible outputs while CoT enabled step-by-step reasoning. Similarly, in the present study, the IPC method was employed to obtain categorical and reproducible outputs, while the CoT approach was used to examine the model’s step-by-step reasoning process [15,33]. In particular, for the visually based London Atlas method, the CoT approach is thought to have contributed to generating results closer to CA by enabling the model to systematically assess reference image comparisons within a structured reasoning chain and to perform similarity–difference evaluations. By contrast, the Nolla and Haavikko methods are based on the numerical scoring of dental mineralization stages rather than direct visual matching. We hypothesize that ChatGPT-4 increased its margin of error in these methods because it attempted to match the scoring table by interpreting the descriptions rather than “measuring” them mentally. The advantage of the CoT approach in visually based comparison methods is thought to be limited in decision-making processes that rely on more abstract and textual criteria instead of precise visual references.

Despite the limited literature on the radiographic image evaluation capabilities of LLMs, a recent study by Camlet et al. [34] compared the performance of different ChatGPT models with that of clinicians in measuring remaining bone height and counting teeth on panoramic radiographs. The authors reported that, while the models demonstrated moderate-to-substantial agreement with clinicians in tooth counting, they consistently overestimated bone height measurements, rendering them unsuitable for clinical application. In another study, Salmanpour et al. [15] reported low accuracy of ChatGPT-4 in panoramic radiographs (37.1% for localization and 46.0% for resorption detection), concluding it was insufficient for clinical use. The lower accuracy in canine impaction tasks likely reflects the need to evaluate multiple anatomical factors simultaneously, which is challenging for a model without domain-specific training. In contrast, dental age estimation provides a more structured process with explicit criteria, allowing ChatGPT-4 to perform better in visually based methods, while its accuracy decreases in scoring-based approaches that rely on textual interpretation.

In forensic applications, the DA should be estimated as accurately as possible. The closer the difference between the CA and DA is to zero, the more accurate any DA estimation method is considered to be. Methods with a mean prediction error below 1 year are also considered accurate [35]. In the present study, ChatGPT-4 achieved MAE values of 0.59 years for boys and 0.60 years for girls when applied using the London Atlas method, both of which fall within generally accepted accuracy thresholds. Orthodontist estimations with the same method yielded an MAE of 0.98 years for girls, also within the acceptable range, but slightly exceeded the threshold for boys (1.19 years). Conversely, ChatGPT-4’s performance with the Nolla method (MAE = 1.42 years for boys; 1.24 years for girls) and the Haavikko method (MAE = 1.17 years for boys; 1.47 years for girls) did not meet the <1-year accuracy criterion, indicating reduced applicability. Orthodontists’ estimations using the Nolla method remained within the threshold for both sexes, whereas their Haavikko method estimations exceeded it. In contrast, in orthodontic and pediatric dentistry contexts, even narrower thresholds may be considered clinically meaningful. For example, Bakhsh et al. [36] proposed a clinically acceptable range of ±0.25 years from the actual CA, as agreed by the principal investigators (orthodontist and pediatric dentist) in their study. According to this stricter criterion, the sub-year differences observed in our study—particularly the 0.59–0.60-year MAE achieved by ChatGPT-4 with the London Atlas method—would still be regarded as clinically acceptable, whereas the higher error rates with the Nolla and Haavikko methods may limit their reliability in routine clinical use.

These findings indicate that the accuracy of ChatGPT-4 is method-dependent, with superior performance in visually guided approaches such as the London Atlas method. Beyond the scope of this study, the results highlight the potential of LLMs as complementary tools in dental age estimation. This suggests that while current performance does not yet surpass deep learning-based image analysis systems, multimodal LLMs may serve as a complementary approach that enhances transparency and reduces examiner subjectivity, representing a promising avenue for future integration into dental and forensic workflows. It is also important to note the forensic and legal implications of dental age estimation. While our findings suggest that multimodal AI systems such as ChatGPT-4 can support DA estimation, these tools remain unvalidated for forensic decision-making. Over-reliance on AI outputs without sufficient validation could pose risks, and such approaches should therefore be regarded as adjuncts rather than replacements for established examiner-based or deep learning-based methods until further evidence is available. It should also be noted that ChatGPT-4 is a general-purpose language model without domain-specific training in dentistry, which may limit the generalizability of its radiographic performance despite the promising results observed with the London Atlas method.

This study has certain limitations that should be considered when interpreting the findings. First, the sample was restricted to a specific geographic region within Turkey, which may limit the generalizability of the results to populations with different genetic, environmental, or socioeconomic backgrounds. However, as the primary objective of this study was to compare the assessments of orthodontists and ChatGPT-4, this limitation may be of lesser concern. ChatGPT-4’s image interpretation was based on a specific preprocessing protocol and presentation format used in this study; variations in image quality, resolution, or display parameters could potentially influence the model’s performance. In addition, the absence of independent assessors among the orthodontist evaluators should be acknowledged as a limitation. Furthermore, future studies should explore ChatGPT’s performance when assessed at different time points and by different evaluators to better understand its consistency, adaptability, and robustness across diverse testing conditions.

## 5. Conclusions

The null hypothesis was rejected, as significant differences were observed between ChatGPT-4, orthodontist, and CA estimations depending on the method applied. Using the London Atlas method, ChatGPT-4 produced results closest to the CA for both boys and girls, with no statistically significant differences observed. In the Nolla and Haavikko methods, ChatGPT-4’s estimates were generally lower than the CA; however, orthodontist assessments exhibited similar trends. Across all methods, the correlation between ChatGPT-4 and orthodontist estimates was high, with the strongest agreement achieved in the London Atlas method. Moreover, the mean prediction error for ChatGPT-4 was lowest in the London Atlas method and highest in the Haavikko method. These findings suggest that ChatGPT-4 can achieve comparatively better accuracy in visually guided, comparison-based methods, whereas its performance is more limited in multi-stage scoring approaches.

## Figures and Tables

**Table 1:** Initial and final optimized prompts developed for dental age estimation with ChatGPT-4.

Prompt Development Stages	Nolla Method	Haavikko Method	London Atlas Method
Initial prompt	“Estimate the DA using the Nolla method.”	“Estimate the DA using the Haavikko method.”	“Estimate the DA using the London Atlas method.”
Optimized prompt after IPC	“I am an orthodontist conducting an ethically approved research study on DA estimation using panoramic radiographs. You will be provided with images and asked to estimate the individual’s DA using the Nolla method. Please apply expert-level reasoning and provide specific, step-by-step evaluations based on the selected method. Identify the seven permanent teeth in the lower left quadrant (excluding the third molar). Assign each tooth a stage from 1 to 10 based on Nolla’s classification. If the development is between stages, use decimal values (e.g., 6.2 or 6.7).”	“I am an orthodontist conducting an ethically approved research study on DA estimation using panoramic radiographs. You will be provided with images and asked to estimate the individual’s DA using the Haavikko method. Please apply expert-level reasoning and provide specific, step-by-step evaluations based on the selected method. Evaluate the appropriate permanent teeth based on the individual’s age group. Assign a stage from 1 to 12 according to Haavikko’s radiological development stages.”	“I am an orthodontist conducting an ethically approved research study on DA estimation using panoramic radiographs. You will be provided with images and asked to estimate the individual’s DA using the London Atlas method. Please apply expert-level reasoning and provide specific, step-by-step evaluations based on the selected method. Evaluate the development and eruption status of all visible permanent teeth and compare the findings to the London Atlas diagrammatic age stages. Choose the closest matching schematic based on visual assessment.”
Final optimized prompt	“For each of the seven teeth, describe the crown and root formation observed in the panoramic image. Justify the stage assigned using Nolla’s criteria. After assigning stages, calculate the sum and divide by the number of evaluated teeth to get the average. Convert this score into DA using Nolla’s reference table and conclude with the estimated age and reasoning.”	“For each selected tooth, describe the degree of crown and root formation and apex closure. Determine the appropriate developmental stage using Haavikko’s criteria. Convert each stage into the corresponding age using the sex-specific table. Average the ages of all evaluated teeth and provide the final DA with reasoning.”	“Analyze the panoramic image for overall tooth development patterns, including crown completion and eruption levels. Compare these to the atlas diagrams. Explain which atlas stage most closely resembles the image, and report that stage’s corresponding age as the estimated DA, with a justification.”

DA: Dental age, IPC: Iterative Prompt Calibration.

**Table 2:** Comparison of DA, DA–CA difference, and MAE by method and evaluator.

CA Mean (SD)	Method	Evaluator	DA Mean (SD)	DA–CA Years Mean (SD)	MAE Years Mean (SD)	*p*-Value
12.37 (2.95)	London Atlas	Orthodontist	13.15 (3.21)	0.78 (1.26)	1.09 (1)	<0.001
		ChatGPT-4	12.41 (2.81)	0.03 (0.93)	0.59 (0.72)	0.399
	Nolla	Orthodontist	12.40 (3.08)	0.03 (1.14)	0.86 (0.75)	0.606
		ChatGPT-4	12.00 (2.57)	−0.40 (1.96)	1.33 (1.28)	<0.001
	Haavikko	Orthodontist	11.49 (2.15)	−0.88 (1.49)	1.29 (1.16)	<0.001
		ChatGPT-4	11.19 (2.07)	−1.18 (1.70)	1.51 (1.41)	<0.001

Paired *t*-test was used to compare DA and CA within each evaluator group. DA: Dental age, CA: chronological age, MAE: mean absolute error.

**Table 3:** Comparison of DA and CA by sex, method, and evaluator.

Sex	CA Mean (SD)	Method	Evaluator	DA Mean (SD)	DA–CA Years Mean (SD)	MAE Years Mean (SD)	*p*-Value
Boys (*n* = 263)	12.39 (2.94)	London Atlas	Orthodontist	13.19 (3.38)	0.80 (1.49)	1.19 (1.20)	<0.001
			ChatGPT-4	12.41 (2.80)	0.02 (0.96)	0.59 (0.76)	0.749
		Nolla	Orthodontist	12.38 (3.03)	−0.01 (1.20)	0.92 (0.77)	0.897
			ChatGPT-4	12.04 (2.38)	−0.34 (1.89)	1.42 (1.30)	0.003
		Haavikko	Orthodontist	11.80 (2.22)	−0.59 (1.38)	1.11 (1.0)	<0.001
			ChatGPT-4	11.52 (2.32)	−0.87 (1.40)	1.17 (1.16)	<0.001
Girls (*n* = 248)	12.35 (2.96)	London Atlas	Orthodontist	13.11 (3.03)	0.79 (1.06)	0.98 (0.72)	<0.001
			ChatGPT-4	12.40 (2.83)	0.05 (0.91)	0.60 (0.68)	0.369
		Nolla	Orthodontist	12.42 (3.15)	0.06 (1.08)	0.81 (0.72)	0.353
			ChatGPT-4	11.96 (2.78)	−0.39 (1.73)	1.24 (1.26)	<0.001
		Haavikko	Orthodontist	11.16 (2.03)	−1.19 (1.54)	1.84 (1.51)	<0.001
			ChatGPT-4	10.86 (1.71)	−1.49 (1.86)	1.47 (1.27)	<0.001

Paired *t*-test was used to compare DA and CA within each evaluator group. DA: dental age, CA chronological age, MAE: mean absolute error.

**Table 4:** Comparison of DA estimates between the orthodontists and ChatGPT-4.

Method	DA–CA (Orthodontist)	DA–CA (ChatGPT-4)	Mean Difference (Orthodontist vs. ChatGPT-4)	*p*-Value
London Atlas	0.78 (1.26)	0.03 (0.93)	0.75 (1.39)	<0.001
Nolla	0.03 (1.14)	−0.40 (1.96)	0.42 (2.17)	<0.001
Haavikko	−0.88 (1.49)	−1.18 (1.70)	0.31 (1.24)	<0.001

Paired *t*-test was used to compare DA and CA between orthodontist and ChatGPT-4. DA: Dental age, CA: chronological age.

**Table 5:** ICC and Spearman correlation coefficients between DA and CA by evaluator and method.

Method	Evaluator	Statistic	Total
London Atlas	Orthodontist/ChatGPT-4	ICC	0.944
		Spearman r	0.905
Nolla	Orthodontist/ChatGPT-4	ICC	0.850
		Spearman r	0.745
Haavikko	Orthodontist/ChatGPT-4	ICC	0.906
		Spearman r	0.819

DA: Dental age, CA: chronological age

## Data Availability

The raw data supporting the conclusions of this article will be made available by the authors on request.

## Abbreviations

The following abbreviations are used in this manuscript:

DA	Dental age
CA	Chronological age
LLMs	Large language models
AI	Artificial intelligence
ChatGPT	Chat Generative Pretrained Transformer
MAE	Mean absolute error
CoT	Chain of Thought
IPC	Iterative Prompt Calibration
